# Impact of financial support on treatment outcomes of multidrug-resistant tuberculosis: A population-based, retrospective cohort study in Shanghai, China

**DOI:** 10.1016/j.jctube.2024.100500

**Published:** 2024-11-29

**Authors:** Yong Chen, Xin Shen, Yi Zhang, Zheyuan Wu, Biao Xu, Jing Chen, Wei Sha, Xiaoxia Liu, Chenxi Ning

**Affiliations:** a Shanghai Municipal Center for Disease Control and Prevention, Shanghai, China; b Shanghai University of Traditional Chinese Medicine, Shanghai, China; c Department of Epidemiology, School of Public Health, Fudan University, Shanghai, China and Key Laboratory of Public Health Safety (Fudan University), Ministry of Education, China; d Shanghai Key Laboratory of Tuberculosis, Clinic and Research Center of Tuberculosis, Shanghai Pulmonary Hospital, Tongji University School of Medicine, Shanghai, China

**Keywords:** Financial burden, Multidrug-resistant tuberculosis, Social protection, Treatment outcomes, Second-line drugs

## Abstract

**Background:**

To date, the prolonged treatment duration and expensive second-line anti-tuberculosis drugs (SLDs) for multidrug-resistant tuberculosis (MDR-TB) can impose a significant financial burden, which may negatively impact treatment outcomes. This study examines the effect of a subsidy policy on treatment outcomes of MDR-TB patient..

**Methods:**

We collected demographic and drug resistance data of all registered MDR-TB patients between April 2011 and December 2019 in Shanghai, China. Documentation of financial support received was routinely maintained until December 2021. We employed multivariate logistic regression to assess the association between financial support and treatment outcomes, estimating odds ratios (ORs) and their corresponding 95% confidence intervals (CIs).

**Results:**

Of the 865 patients, 70.6% (611/865) achieved treatment success. The median amount compensated under the subsidy policy was 2359 United States dollar (USD), with an interquartile range from 1116 to 5652 USD. A positive association was found between benefiting from the subsidy policy and higher rate of treatment success, with an adjusted OR of 2.95 (95% CI, 2.03–4.28). Among the 641 patients covered by the policy, the adjusted OR comparing those with higher versus lower reimbursement was 1.74 (95% CI, 1.16–2.61).

**Conclusions:**

Financial support policies for MDR-TB patients demonstrate a positive influence on treatment outcomes.

## 1 Introduction

Multidrug-resistant tuberculosis (MDR-TB) remains a global public health concern. Recent data show that the global treatment success rate of MDR-TB is only 63 % [1]. In most cases, MDR-TB medical care requires regimens containing multiple second-line anti-tuberculosis drugs (SLDs) across a prolonged treatment duration. Although lifesaving, most MDR-TB regimens are many times more expensive than those for drug-susceptible TB [2], [3]. For example, the average cost of MDR-TB treatment in Estonia and Russia was about 10,000 USD in the early years [4]. A study in South Africa suggested that approximately 70 % of drug-resistant TB costs were attributed to anti-TB drugs and hospitalization [5]. In Germany and the United States, the costs were even higher [6], [7].

Consequently, MDR-TB patients often suffer high out-of-pocket (OOP) expenditures [8]. A systematic review reported that 80 % of MDR-TB patients face catastrophic costs [9]. In low-and middle-income countries, the per-patient cost is relatively lower, but still enough to incur high proportion of catastrophic costs [5]. In 2018, the updated WHO guidelines [10] introduced novel drugs (bedaquiline and delamanid) and increased the importance of linezolid and clofazimine in the recommended regimens. This improvement, however, increased the already heavy costs of treatment, creating further financial barriers to accessing affordable treatment. Several novel short-term regimens were also introduced recently [11], with emerging evidence suggesting that these shorter regimens may offer significant cost savings due to improved therapeutic efficacy and reduced treatment duration [12], [13]. However, most of these regimens remain under investigation in clinical trials. Recent data suggest that the pooled average percentage of drug-resistant TB patients facing catastrophic costs was 83 % [1], and was much higher in countries such as Vietnam [14].

China currently bears the burden of the world's second highest MDR-TB caseload. However, the treatment success rate for MDR −TB in China is approximately 51 % [15]. Chinese government funding for TB control, which consists of the national TB Program, basic health insurance, and varied regional reimbursement settings, has increased in recent years. However, the existing TB program does not encompass certain essential SLDs, and these medications are also absent from the health insurance program. Consequently, the costs for treating MDR-TB lack financial security [16]. The estimated cost of individualized treatment regimens could reach 9000 USD in China, whereas the support package for MDR-TB care was only 4644 USD in 2015[17]. Furthermore, patients frequently incurred additional expenses related to transportation and accommodation while also experiencing indirect costs associated with wage loss. The combination of both direct medical and non-medical expenditures, along with indirect costs, has resulted in significant financial hardship [2], [18], [19]. Previous studies suggested that the median OOP payment of patients with MDR-TB in China might exceed 3500 USD [20], and approximately 80 % of patients incurred catastrophic expenditures even with health insurance [21]. This financial barrier might lead to interruptions in receiving timely, appropriate medical care, poor adherence to treatment, or loss to follow-up [2]. Missing doses and inappropriate use of drugs could contribute to the development of severe disease, amplified drug resistance, and treatment failure [19], [22], [23], [24]. Meanwhile, prolonged, irregular treatment could potentially cause further transmission of drug-resistant strains [25]. Hence, it is crucial to develop and evaluate targeted financial support measures.

Preliminary studies have indicated that financial protection was related to treatment results of MDR-TB [26], [27], [28]. However, the literature on the impact of quantified financial support on MDR-TB treatment outcomes is scant, particularly in the context of novel anti-TB drugs and short-term regimens introduced in the recent years. This study aimed to quantitatively assess the impact of a tailored subsidy policy for MDR-TB patients, and to determine whether the policy positively influenced treatment outcomes, providing evidence for the development and improvement of similar policies.

## 2 Methods

### 2.1 The subsidy setting for MDR-TB

In the recent years, Shanghai’s annual TB incidence rate is approximately 20/100,000, with an estimated 100–150 confirmed MDR-TB cases annually. A population-based registry of confirmed patients with MDR-TB has been established in Shanghai, China since 2011. This comprehensive setting of diagnosis, treatment, and management was described previously [29]. The National Basic Public Health Service funds the provision of patient management in communities. Basic health insurance typically covers the costs of regular treatment with first-line drugs, whereas certain SLDs are not covered.

**Fig. 1:**
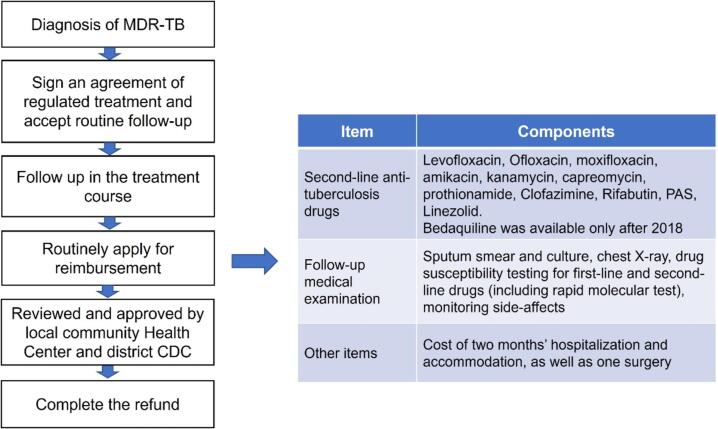
Procedure and coverage of the subsidy policy for MDR-TB treatment in Shanghai, China.

To mitigate the financial burden of MDR-TB healthcare services, Shanghai introduced a subsidy scheme initially targeting anti-TB drugs, which gradually expanded to MDR-TB medical care costs that were not included by the basic health insurance since 2011. Funding of this policy was the integration of national TB program and local government health expenditure. Starting from 2018, the policy further expanded, covering SLDs such as linezolid and bedaquiline. In addition, the subsidy package provided compensation for two months of hospitalization and surgery expenses, along with monthly follow-up visits and examinations [30] (Fig. 1). The subsidy procedure required patients to enter into agreements with local disease control agencies (district centers for disease control and prevention, CDCs) and community health centers, committing to regular medication intake and follow-up visits as necessary. Patients should pay OOP for medical service initially, then apply for reimbursement by submitting receipts. This process involved review and approval by healthcare workers in local community health centers and district CDCs. The subsidy policy operated alongside the existing basic health insurance.

### 2.2 Data collection

All patients diagnosed with MDR-TB in Shanghai from April 2011, when the MDR-TB registry in the Shanghai Center for Disease Control and Prevention (SCDC) was initiated, and December 2019 was collected. Among the 1016 patients enrolled, 151 who were still in treatment by the end of 2021 were excluded, leaving 865 patients for analysis. Information on demographics and treatment history of the patients were collected from the MDR-TB registry. Resistance to SLDs was assessed to identify extensively drug-resistant TB (XDR-TB) and pre-XDR-TB at SCDC and designated MDR-TB hospitals using the isolated *M. tuberculosis* strains.

The primary outcome in this study was the treatment results, which were ascertained by linkage to the National TB registry database. The definition of treatment outcomes was in accordance with the WHO guidelines [31]. We further defined treatment success of MDR-TB as cured or treatment completion, whereas adverse outcomes referred to treatment failure, death, loss to follow-up, or unknown outcome. Of the 865 patients, 313 were classified as cured and 298 had completed their treatment. These numbers correspond to 611 patients categorized as treatment success, accounting for 70.6 % of the cohort. Eighty-seven the patients were recorded as treatment failure (10.1 %), and 75 died during treatment (8.7 %). Forty-eight patients were recorded as loss to follow-up (5.6 %), whereas 44 were categorized as having unknown outcomes (5.1 %). Thus, a total of 254 patients (29.4 %) had adverse outcomes.

The district CDCs systematically documented subsidy information, which was subsequently reported to SCDC. These records exclusively included data from patients who had signed the requisite agreements. The reimbursement coverage included OOP for various expenses, such as laboratory test, follow-up medical examination, anti-TB medicines, liver-protective drugs, hospitalization, and surgery. Meanwhile, the amount of health insurance received during the same period was recorded for patients who applied for reimbursement. All financial support data, denominated in CNY, were collected by the end of 2021 and converted to USD in this study, based on an exchange rate of 1 USD to 6.37 CNY as of December 2021.

### 2.3 Statistical analyses

Differences in demographic and drug-resistant patterns for patients with treatment success and those with adverse outcomes were treated as categorical variables and were evaluated using χ^2^ tests. The amount of reimbursed financial support was compared between different groups using non-parametric tests. We performed multivariate logistic regression to estimate the association between the situation of subsidies (received/not received any financial support) and outcomes by estimating odds ratios (ORs) and 95 % confidence intervals (CIs). For patients who received any financial support, we further employed multivariate logistic regression to estimate whether greater amount of financial support was associated with treatment success. In these models, we used the median of financial support as cutoff points to define greater/less reimbursement as a categorical variable. The OR estimates in the regressions were adjusted for potential confounders including age, sex, being a local resident, before/after the expanded subsidy policy in 2018, and previous TB treatment history. In sensitivity analyses, drug-resistance pattern was further adjusted. We applied the restricted cubic spline (RCS) function in logistic models to quantitively demonstrate the association pattern. In these analyses, financial support was treated as continuous variable, while knots were placed at the 10th, 50th, and 90th percentiles, and the 50th percentiles were used as the reference [32]. Statistical analyses were conducted using SAS 9.4 (SAS Institute Inc., Cary, NC, USA). R 4.1.3 was used to plot the RCS curves. All tests were 2–sided and considered statistically significant at *P* < 0.05.

## 3 Results

Basic demographic and treatment profiles of the patients are presented in Table 1. The mean (standard deviation) age of the patients at diagnosis was 43.7 (16.7) years. Approximately 70 % of the patients were male and local residents in Shanghai. Nearly half of the patients were previously treated. At MDR-TB diagnosis, 740 patients had profiles of resistance to SLDs, of which 225 were pre-XDR-TB and 82 were XDR-TB. Older age, covered by the expanded subsidy policy in 2018, female gender, and patients from areas other than Shanghai were more likely to have treatment success. Among those had information on drug-resistance results, pre-XDR-TB (59.1 %) and XDR-TB (56.1 %) patients had lower rate of treatment success. Among 611 patients with treatment success, 478 (78.2 %) benefited from the subsidy policy, whereas 163 of 254 patients (64.2 %) with adverse outcomes received financial support.

**Table 1:** Characteristics of MDR-TB patients in Shanghai, China, from 2011 to 2019, by treatment outcomes.

Characteristic	Overall (*n* = 865)			Treatment success (*n* = 611)			Adverse outcome (*n* = 254)		*P* value
	No.	%		No.	%		No.	%	
Age at MDR-TB diagnosis									
<30 years	237	27.4		205	86.5		32	13.5	<0.001^a^
30–60 years	463	53.5		309	66.7		154	33.3	
>60 years	165	19.1		97	58.8		68	41.2	
Underwent the expanded subsidy policy									
No (registered before May 2018)	630	72.8		419	66.5		211	33.5	<0.001
Yes (registered after May 2018)	235	21.2		192	81.7		43	18.3	
Sex									
Male	620	71.7		414	66.8		206	33.2	<0.001
Female	245	28.3		197	80.4		48	19.6	
Residential area									
Locals in Shanghai	601	69.5		391	65.1		210	34.9	<0.001
From other provinces	264	30.5		220	83.3		44	16.7	
TB treatment history									
New cases	438	50.6		351	80.1		87	19.9	<0.001
Previously treated	427	49.4		260	60.9		167	39.1	
Drug susceptibility *									
MDR-TB only	433	50.1		334	77.1		99	22.9	<0.001^b^
Pre-XDR-TB	225	26.0		133	59.1		92	40.9	
XDR-TB	82	9.5		46	56.1		36	43.9	
Not tested for SLDs	125	14.4		98	78.4		27	21.6	

MDR-TB: multidrug-resistant tuberculosis; XDR-TB: extensively drug-resistant tuberculosis; SLDs: second-line anti-tuberculosis drugs.

a Mantel-Haenszel χ^2^ test.

b Mantel-Haenszel χ^2^ test. The *P* value was calculated among 740 patients with available drug-resistance profiles at MDR-TB diagnosis.

The total financial support reimbursed under the subsidy policy during the study period amounted to 2.62 million USD. Anti-TB drugs accounted for most of the subsidy scheme, totaling 2.20 million USD (83.8 %), followed by laboratory test/follow-up examinations at 0.23 million USD (8.7 %). The remaining reimbursement covered hospitalization, surgery, and liver-protective medications. The median reimbursement under the subsidy policy received was 2359 USD (Table 2). In total, patients with treatment success received greater amount of financial support under the policy compared to those with adverse outcomes (median, 2800 USD vs. 1538 USD, P < 0.001). This difference was consistent when considering health insurance compensation. Similarly, the average monthly financial support differed significantly between these two groups (128 USD vs. 74 USD, P < 0.001).

**Table 2:** Financial support received among MDR-TB patients in Shanghai, China, by treatment outcomes, USD.

Financial support	Overall			Treatment success			Adverse outcomes		*P* value a
	*n*	Median [IQR]		*n*	Median [IQR]		*n*	Median [IQR]	
Diagnosis and follow-up examination	641	279 [144–464]		478	306 [178–486]		163	194 [94–377]	<0.001
Anti-tuberculosis drugs	641	1711 [555–4767]		478	2142 [701–5469]		163	894 [298–2193]	<0.001
Liver-protect drugs	641	96 [0–276]		478	120 [0–291]		163	79 [0–204]	0.347
Hospitalization and surgery	641	24 [0–132]		478	24 [0–126]		163	20 [0–141]	0.647
Total financial support under the subsidy policy	641	2359 [1116–5652]		478	2800 [1330–6441]		163	1538 [618–2799]	<0.001
Combined financial support of the subsidy policy and health insurance	433	4092 [1738–9210]		319	5271 [2030–9557]		114	2673 [974–6198]	<0.001

IQR: interquartile range.

a Kruskal-Wallis test.

Overall, 77.9 % of patients under the subsidy policy had treatment success. In contrast, the treatment success rate for those who did not benefit the subsidy policy was 64.3 %. In the multivariable logistic models, covered by the policy was positively associated with better treatment success, with an adjusted OR equal to 2.95 (95 %CI 2.03–4.28). This association did not differ by the expansion of the subsidy policy in 2018. Among 740 patients who had drug resistance profiles, this positive association was also evident (OR = 3.21, 95 %CI 2.10–4.90). Moreover, multivariate regression analyses suggested that patients who received higher amount of financial assistance had better treatment outcomes. Among 641 patients covered by the policy, the OR comparing those who received greater versus less reimbursement was 1.74 (95 % CI 1.16–2.61). After further adjustment for drug resistance pattern, the association remained significant (OR = 2.12, 95 % CI 1.36–3.32). Among patients with information on health insurance compensation, the significant association was still observed (Table 3).

**Table 3:** Association between financial support reimbursed and treatment success.

Financial assistance	Treatment success in model 1				Treatment success in model 2		
	*n* (%)	OR (95 % CI) a	*P* value		*n* (%)	OR (95 % CI) b	*P* value
Reimbursement under the subsidy policy (USD)							
≤2359	212(66.0)	Reference			169(63.8)	Reference	
>2359	266(83.1)	1.74(1.16–2.61)	0.008		239(81.9)	2.12(1.36–3.32)	0.001
Combined reimbursement of the subsidy policy and health insurance (USD)							
≤4093	145(67.1)	Reference			116(64.8)	Reference	
>4093	174(80.2)	1.35(0.83–2.19) c	0.231		155(79.1)	1.73(1.01–2.94) d	0.045

a Adjusted for age, sex, residential area, TB history, and covered by the expanded subsidy policy in 2018; 641 patients were included in this model.

b Further adjusted for drug resistance pattern (categorical: MDR only, pre-XDR, and XDR); 557 patients with available drug resistance profiles were included.

c 433 patients with health insurance profiles were included.

d 375 patients with health insurance profiles and drug-resistance information were included.

**Fig. 2:**
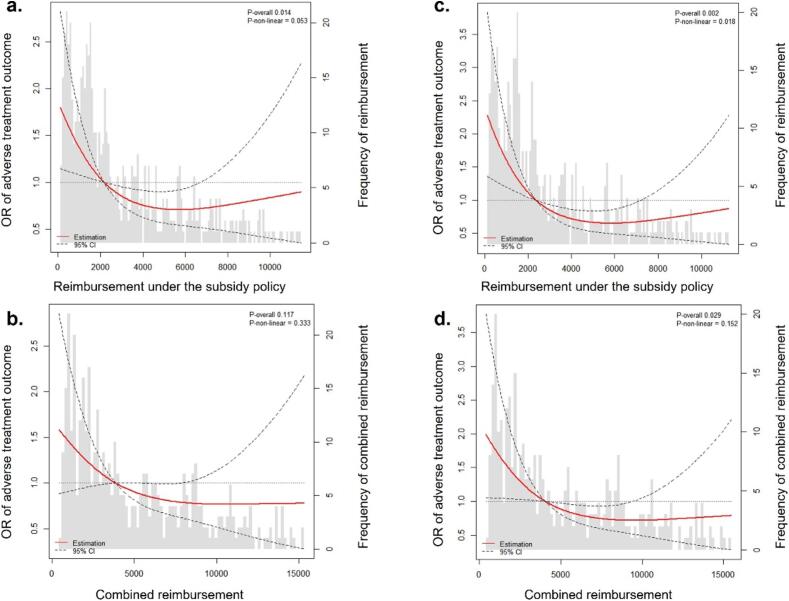
Association between the amount of financial support and treatment outcomes using a restricted cubic spline regression model. Results were adjusted for age, sex, residential area, TB treatment history, and covered by the expanded subsidy policy in 2018 (a,b) and further adjusted for drug resistance pattern (c,d). A restricted cubic spline regression model was conducted with three knots at the 10th, 50th, and 90th percentiles of the amount of reimbursement. The dotted lines represent the 95% confidence intervals for the spline model (the reference was the 50th percentile). The shaded histograms represent the distribution of the amount of reimbursement. Those who received financial support greater than the 95th percentile were not included in these models, for there were too few data points with very high reimbursement.

After adjusting for potential confounders, RCS showed an L-curved association pattern between the amount of financial support received and lower risks of adverse outcomes (Fig. 2). The risks kept decline as financial support increased, until the reimbursement reached around 6000 USD (or around 9000 USD when health insurance was considered). After these points, this association seems to level off.

## 4 Discussion

In this retrospective study encompassing all registered MDR-TB patients in a megacity, the extensive subsidy strategy was comprehensively assessed. To our knowledge, this is the first study to provide quantitative evidence demonstrating a positive association between financial support and improved MDR-TB treatment outcomes. The population-based study design, encompassing over 800 patients across nine years, ensured sufficient statistical power. Furthermore, the well-established MDR-TB control network in Shanghai [29] facilitated the consideration of covariates, mitigating potential biases. The findings underscore the potential of financial interventions to not only advance the End TB strategy by bridging the gap between catastrophic expenses and household finances [33], but also to yield direct, clinical beneficial improvements. Our investigation revealed that nearly three-quarters of the patients in our cohort benefited from the subsidy policy, which served as an important addition to the national basic health insurance. Our previous study documented that the median cost of MDR-TB treatment in Shanghai was 4961 USD before 2014. Another study in eastern China reported a median direct cost of 3727 USD. In Southeast Asia, the average treatment costs for long regimens were 2695.58 USD in Vietnam and 3677 USD in Philippines [34], [35]. Therefore, Shanghai’s subsidy policy, with a combined median reimbursement of 2800 USD, is estimated to cover approximately 70 % of the direct medical costs of MDR-TB treatment, which might largely alleviate the financial hardship and reducing insufficient utilization of treatments and follow-up services.

Our analysis revealed an independent association between financial assistance and treatment outcomes. Additionally, a robust link between reimbursement magnitude and treatment success was observed. The treatment outcomes of MDR-TB patients are influenced by a complex interplay of behavioral, socioeconomic, clinical, and healthcare service risk factors [36], [37]. Lower socioeconomic status is often related to financial hardship of regulated treatment, poorer nutritional health, and social stigma. Our previous research has demonstrated that lower household income and limited health insurance coverage are associated with catastrophic expenditures [38]. Financial protection from sustained, sufficient subsidies could potentially mitigate financial hardship by reducing OOP payments, thereby increasing patients’ accessibility and affordability to appropriate medical services [25]. The ameliorated outcomes may be ascribed to heightened compliance, attenuating the risks of adverse effect and curtailing amplification of drug resistance. This hypothesis is corroborated by research in Peru, Vietnam, Thailand, and Indonesia [19], [22], [27], [39], presenting evidence of patients with health insurance or financial aid experiencing reduced OOP expenses and superior sputum conversions. Meanwhile, adverse events are common during long-term treatment of more toxic SLDs, potentially leading to interruptions and treatment failure [40]. In our observation, reimbursement for liver protection medications might reduce the risks of adverse events as well.

In the models with the RCS function, we observed that patients with very high reimbursement (approximately over 6000 USD) did not exhibit better outcomes compared to those who received moderate financial assistance. This group typically underwent longer treatment courses and had worse prognoses due to complicated, expensive treatment, lower drug tolerance and amplified resistance [41]. Therefore, specific support programs based on individual regimens and clinical profiles might be considered for these patients.

The current subsidy policy presents opportunities for improvement. Notably, around a quarter of the patients, particularly non-local residents, exhibited lower participation rates in the policy. These patients were younger (median age, 30.5 years vs. 50.0 years, P < 0.001) and tended to seek advanced treatment options in Shanghai. However, they might encounter challenges in the submission-review-approval process, potentially due to misplaced/lost receipts and difficulties with transportation and accommodations. Therefore, enhancing the accessibility and streamlining of the process is crucial for maximizing the policy’s reach and effectiveness. Secondly, the breadth of the subsidy policy might be inadequate compared to the outlay on novel MDR-TB regimens. A survey in 2017 indicated that the average OOP expenditure of MDR-TB patients in China approximated to 10,989 USD [16]. We extrapolated the newly recommended 18-month regimen incorporating bedaquiline and linezolid in Shanghai to approximately 18,838 USD for medications alone. Yet, the median reimbursement we observed was mere 3504 USD after 2018.

A notable limitation in this study was the lack of individual and household financial situations, which was not included in the MDR-TB registry. Furthermore, calculating precise direct medical costs posed difficulties due to varied, frequent modifications of medications. Some patients lacked clear information on their health insurance coverage, complicating the determination of exact OOP expenditure. Additionally, we did not perform a systematic data collection on the quality of patient management. Given that adherence and financial hardship were potential mediators, we omitted these variables from regression analyses to concentrate on understanding the total effect of financial support on treatment outcomes.

## 5 Conclusion

Our study indicate that Shanghai’s subsidy policy mitigates the financial hardship of MDR-TB, irrespective of basic health insurance coverage, positively influencing patients’ treatment outcomes. The results underscore the importance of universally accessible and expanded financial assistance programs for MDR-TB patients. Further studies investigating various TB service delivery settings and social protection mechanisms are merited to evaluate the cost-effectiveness and health impact of financial support measures, particularly with the introduction of novel anti-TB medications.

E**thical approval and consent statement**.

This study was approved by the Institutional Review Board at Shanghai Municipal Center for Disease Control and Prevention in May 2021 (2021–60). No informed consent was sought because only anonymized secondary data in registry was used.

Funding source.

This work was supported by the National Natural Science Foundation of China [81872679 to SHEN Xin] and Shanghai Three-year Action Plan to Strengthen the Public Health System [GWVI-9 to SHEN Xin], as well as the Shanghai Health Commission [202140478 to CHEN Yong]. The sponsors of the study had no role in study design, data collection, data analysis, data interpretation, or writing of the manuscript.

## CRediT authorship contribution statement

**Yong Chen:** Writing – review & editing, Writing – original draft, Visualization, Validation, Supervision, Project administration, Methodology, Investigation, Funding acquisition, Formal analysis, Data curation, Conceptualization. **Xin Shen:** Writing – review & editing, Writing – original draft, Visualization, Validation, Supervision, Project administration, Methodology, Investigation, Funding acquisition, Formal analysis, Data curation, Conceptualization. **Yi Zhang:** Writing – review & editing, Writing – original draft, Visualization, Validation, Supervision, Project administration, Methodology, Investigation, Funding acquisition, Formal analysis, Data curation, Conceptualization. **Zheyuan Wu:** Writing – review & editing, Formal analysis, Data curation. **Biao Xu:** Writing – review & editing, Validation, Methodology, Investigation, Conceptualization. **Jing Chen:** Writing – review & editing, Data curation, Conceptualization. **Wei Sha:** Writing – review & editing, Investigation, Formal analysis, Data curation. **Xiaoxia Liu:** Writing – review & editing, Formal analysis, Data curation. **Chenxi Ning:** Writing – review & editing, Formal analysis, Data curation.

## Declaration of competing interest

The authors declare that they have no known competing financial interests or personal relationships that could have appeared to influence the work reported in this paper.
